# Clinical Significance of Procalcitonin, C-Reactive Protein, and Interleukin-6 in Helping Guide the Antibiotic Use for Patients with Acute Exacerbations of Chronic Obstructive Pulmonary Disease

**DOI:** 10.1155/2021/8879401

**Published:** 2021-03-15

**Authors:** Wen Song, Yue Wang, Fengming Tian, Liang Ge, Xiaoqian Shang, Qiang Zeng, Ning Feng, Jiahui Fan, Jing Wang, Xiumin Ma

**Affiliations:** ^1^State Key Laboratory of Pathogenesis, Prevention and Treatment of High Incidence Diseases in Central Asia, Clinical Laboratory Center, Tumor Hospital Affiliated to Xinjiang Medical University, Urumqi, 830000, China; ^2^First Affiliated Hospital of Xinjiang Medical University, Urumqi, Xinjiang 830011, China; ^3^The Seventh Affiliated Hospital of Xinjiang Medical University, Urumqi, Xinjiang 830011, China; ^4^Respiratory Department of the Second Affiliated Hospital of Hainan Medical College, Haikou, Hainan 570000, China

## Abstract

**Background:**

Currently, standards of antibiotic use in acute exacerbations of chronic obstructive pulmonary disease (AECOPD) patients are controversial.

**Objective:**

The aim of the present study was to analyze the value of procalcitonin (PCT), C-reactive protein (CRP), and interleukin-6 (IL-6) levels to guide the antibiotic treatment of AECOPD patients.

**Methods:**

A total of 371 patients with COPD or AECOPD were included in the study. Clinical and laboratory data were obtained at admission, 325 AECOPD patients and 46 sCOPD patients treated with antibiotics. The receiver operating curve (ROC) was used to evaluate the relationship between CRP, PCT, and IL-6.

**Results:**

This study included medical record/case control 1, the COPD group (*n* = 46) and the AECOPD group (*n* = 325), and medical record control 2, the nonchanged antibiotic group (*n* = 203) and the changed antibiotic group (*n* = 61). In case 1, CRP, PCT, and IL-6 levels in the AECOPD group were higher than that in the control group (*P* < 0.05), while the result of ROC showed that IL-6 had higher AUC values (0.773) and higher sensitivity (71.7%) than other indicators. The specificity of PCT (93.5%) is higher than other indicators. In case 2, ROC curve results showed that the AUC value of IL-6 (0.771) was slightly higher than PCT and CRP. The sensitivity (85.2%) and specificity (65.5%) of CRP were higher than other indicators.

**Conclusions:**

IL-6 and PCT were elevated in AECOPD patients, resulting in a higher diagnostic value for AECOPD. CRP had a higher diagnostic value for antibiotic use in AECOPD patients.

## 1. Introduction

Chronic obstructive pulmonary disease (COPD) is believed to be caused by multiple factors, virus infection, air pollution, and other factors, that can aggravate airway inflammation, leading to subsequent infection, among which respiratory tract bacterial or virus infection [1–3]. Acute exacerbations are often triggered by the acquisition of new bacterial strains in stable chronic obstructive pulmonary disease (sCOPD) patients; these new bacterial strains can lead to acute exacerbations of chronic obstructive pulmonary disease (AECOPD) with more sensitive airway response and systemic inflammation [4]. Forty-six percent of patients with COPD had at least one exacerbation in the past year, and nineteen percent of patients were even required hospitalization [4]. Most patients with AECOPD were prescribed antibiotics, and the GOLD criteria recommend that antibiotics be used when there were obvious clinical indications, as it could reduce recovery time, the risk of early recurrence and treatment failure, and the length of hospital stay [4]. Actually, the usage rate of patients with AECOPD who received antibiotic treatment was as high as 85%-89% [5, 6]. However, not all patients benefit from antibiotic treatment, and the abuse of antibiotics may increase the risk of bacterial resistance and the waste of resources [7].

The crucial issue of patients with AECOPD is been how to determine who is most likely to benefit from antibiotic treatment. It is non-real-time and not possible for every specimen for evidences of antibiotic treatment. Clinicians come to fully appreciate the fact that laboratory inflammation markers may be useful. C-reactive protein (CRP), procalcitonin (PCT), interleukin-6 (IL-6) are objective and quantitative indicators, which can be quickly completed the detection.

Studies on COPD caused by sulfur mustard have shown the increased CRP and IL-6 during long-term chronic inflammation [8, 9], and when acute exacerbations are also related to lung disease symptoms, not only with the local anti-inflammatory therapy of lung but also the inflammatory system of the patient [10]. Other studies have shown that in COPD patients, CRP and IL-6 have been significantly higher than in healthy people [11]. CRP have a reverse statistically significant relationship with FEV1; IL-6 levels are not significantly correlated with it [12]. PCT is a classic inflammatory marker. The elevated CRP is slightly earlier than PCT, but with a half-life of 19 h. The level of CRP at least 22 mg/L was associated with failure antibiotic therapy in the first 48 h [13]. PCT levels start to rise 6–12 h after the infectious stimulus [13–15]. Supporting evidence came from research showing that PCT and CRP not only have the ability to distinguish and differentiate bacterial respiratory infections in patients with AECOPD but also can be used to guide antibiotic management [14, 16, 17]. There is a relatively slow level of PCT and CRP in AECOPD patients. Therefore, it is necessary to find new quantitative markers. Lorenz Weidhase's study suggested that IL-6 is better than PCT and CRP in predicting the antibiotic therapy success in predominantly nonsurgical sepsis in the first 48–72 h. But there is little information that IL-6 is a guide to antibiotic therapy in patients with AECOPD. IL-6 is one of the most important inflammatory cytokines. It has been shown a significant elevation, not merely as a risk factor in AECOPD patients but as an effective predictor of AECOPD progression [18]. It is secreted by hepatocytes during bacterial infections, which are believed to protect the body from overshooting inflammatory reactions [13]. Inflammation or other stimuli may rise within a few minutes, with a half-life of about an hour. And the elevated level of IL-6 correlated with the extent of the inflammation [13, 19].

It was a retrospective study. The advantages and reliability of CRP, PCT, and IL-6 were analyzed to help the antibiotic use in patients with AECOPD, to reduce antibiotic exposure, decrease the rate of readmission, shorten hospital stays, and depress mortality without increasing the risk of treatment failure.

## 2. Materials and Methods

### 2.1. Patients

A total of 445 patients with COPD and AECOPD were collected from the First Affiliated Hospital of Xinjiang Medical University. There were 371 cases that had enrolled for this study based on defined criteria. AECOPD was defined as deteriorated of respiratory symptoms, such as dyspnea, change in sputum color or character, and increased sputum volume (two symptoms) or one symptom and one mild symptom (wheezing, pharynx, cough, and nasal congestion/runny nose for at least two consecutive days depending on previous treatment). There is no adjustment of long-term therapeutic schemes (including inhaled and oral medications) within the last 14 days [4]. Informed consent has been received from all patients.

In this study, the clinical data of patients are divided into two medical records for analysis. The control group was the sCOPD group, and the case group was the AECOPD group (case control 1). Among them, according to the different antibiotic therapeutic schemes of patients in the AECOPD group, the patients were divided into the nonchanged antibiotic group and the changed antibiotic group (case control 2) [14, 20, 21]. The positive lung CT report indicated inflammation or a pulmonary infection as recorded in the medical record. The patient was examined by an experienced respiratory physician; positive results contained wet rales, dry rales, or sputum purrs during auscultation that indicate positive auscultation.

### 2.2. Inclusion Criteria

The inclusion criteria were as follows: patients with a definite diagnosis of AECOPD or COPD; hospitalized COPD patients in the same period; clinical data of the same patients were collected after multiple hospitalizations; stay in hospital for more than 24 hours; and complete clinical data.

### 2.3. Exclusion Criteria

**Figure 1:**
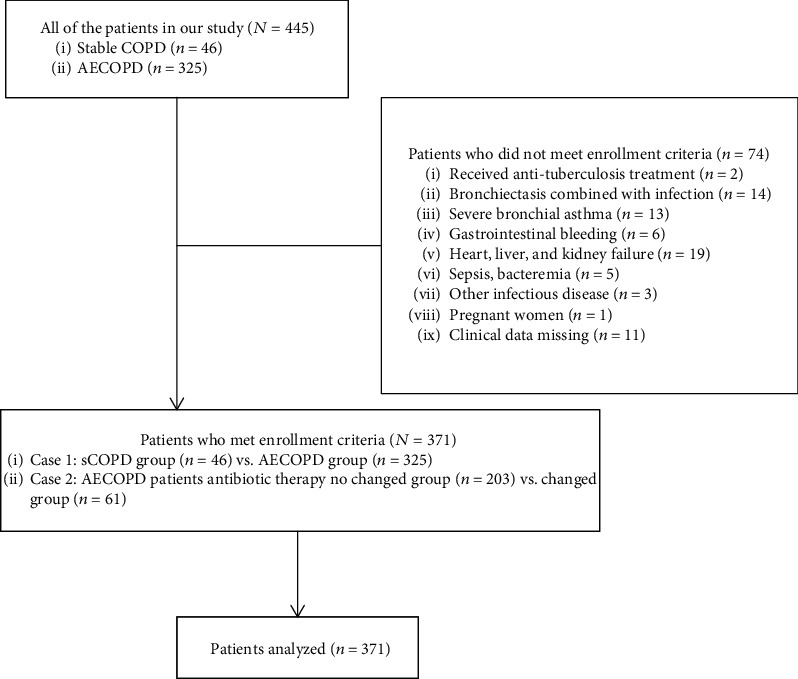
The study involved patients.

The exclusion criteria were as follows: patients received antituberculosis treatment; patients with bronchiectasis combined with infection and bronchial asthma (critical type); patients with gastrointestinal bleeding and severe heart, liver, and kidney failure recently; clear diagnosis of sepsis, bacteremia in patients; patients with other tangible proofs for infectious disease; patients who did not cooperate or had clinical data missing; and pregnant women (Figure 1).

### 2.4. Determination of Biomarkers

Specimens were collected from patients in fasting state in the early morning. Blood samples were collected using heparin lithium anticoagulant vacuum tubes (Becton, Dickinson and Company, USA), centrifuged at 3000 RPM for 10 min. Then, CRP, PCT (Roche Cobas 700, Roche Diagnostics, Mannheim, Germany), and IL-6 (ADVIA Chemistry XPT System, Germany) were detected within 30 minutes.

### 2.5. Statistical Analysis

SPSS V.22.0 software (BMI, Chicago, USA) was used to analyze the data. Compared to the clinical features of AECOPD and sCOPD patients, qualitative and quantitative variables are expressed as count and percentage, mean ± standard deviation. The chi-square test and *T*-test were used to compare the differences between groups. Receiver operating characteristic (ROC) curve analysis was used to research the accuracy of various diagnostic tests. The area under the curve (AUC) and 95% CI for PCT, CRP, and IL-6 were detected to distinguish between patients with/without acute exacerbations of chronic obstructive pulmonary disease. PCT, CRP, IL-6, and antibiotic prescription were predicted by logistic regression analysis. The statistical significance level was set as *P* < 0.05.

## 3. Results

### 3.1. Clinical Data of Patients

The clinical data of patients with COPD and AECOPD was shown in Table 1. The study included 371 patients. There were no statistically significant differences in gender, age, or length of hospital stay in the two groups (*P* > 0.05). In the clinical manifestations of patients' chief complaints, there were statistically significant differences between the two groups in cough, sputum, and purulent sputum (*P* < 0.05). There was no statistical difference between the two groups in the underlying diseases (*P* > 0.05). PCT, CRP, IL-6, pulmonary CT, and pulmonary auscultation were statistically different between the two groups (*P* < 0.05). Compared with the sCOPD group, changed of antibiotics and the addition of antifungal drugs were statistically different in the selection of antibiotic treatment regimens (*P* < 0.05).

### 3.2. Diagnostic Value of PCT, CRP, and IL-6 Levels in Patients with AECOPD

**Figure 2:**
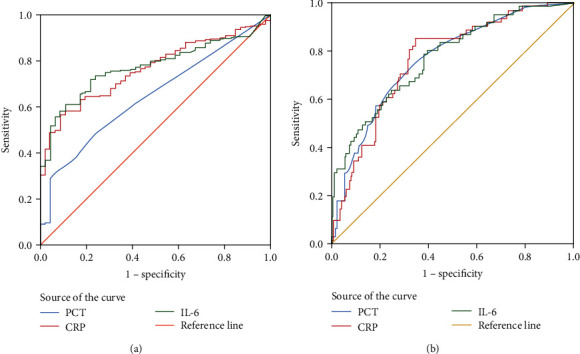
ROC curve of PCT, CRP, and IL-6 levels and their diagnostic value for AECOPD and antibiotic prescription prediction. Note: (a) ROC curve of PCT, CRP, and IL-6 levels and their diagnosis of AECOPD in case 1; (b) the ROC curve of PCT, CRP, and IL-6 levels predicts the application of AECOPD antibiotics in medical record case 2.

For the biomarkers, the peak area under the ROC curve of IL-6 was 0.773 (95% CI: 0.738–0.843; *P* < 0.001) and was higher than CRP (AUC: 0.764; 95% CI: 0.703–0.814; *P* < 0.001) and PCT (AUC: 0.647; 95% CI: 0.573-0.720; *P* < 0.001) to predict AECOPD, respectively (Table 2, Figure 2(a)). When the cut-off value of IL-6 was 5.262, the sensitivity and specificity of IL-6 were 71.7% and 78.3% (Table 2).

### 3.3. The Predictive Value of PCT, CRP, and IL-6 in Antibiotic Prescription

To our surprise, the AUC and specificity of these three indicators were very similar, including PCT (0.764), CRP (0.764), and IL-6 (0.771) (Figure 2(b)). However, the sensitivity of CRP (85.2%) was significantly higher than that of PCT (75.4%) and IL-6 (80.3%). When the CRP level of AECOPD patients was higher than the cut-off value of 20.205 mg/mL, it may be necessary to start antibiotic therapy (Table 2).

**Figure 3:**
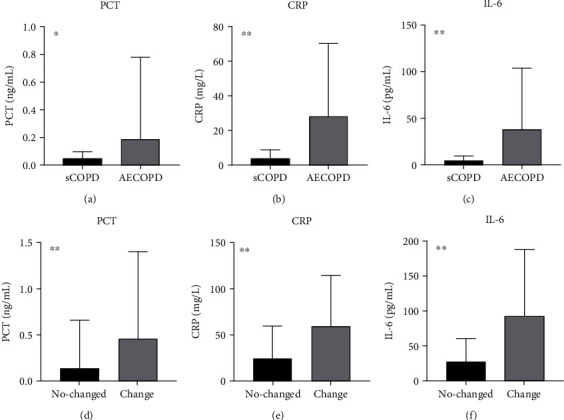
Comparison of PCT, CRP, and IL-6 between case 1 and case 2. Note: (a–c) the comparison of PCT, CRP, and IL-6 in case 1. (d–f) The comparison of PCT, CRP, and IL-6 in case 2. ^∗^*P* < 0.05; ^∗∗^*P* < 0.001.

It indicated that PCT (*P* < 0.05), CRP (*P* < 0.001), and IL-6 (*P* < 0.001) were higher than the sCOPD group (Figures 3(a)–3(c)). Patients with AECOPD those who did not get better with antibiotics showed higher PCT (*P* < 0.001), CRP (*P* < 0.001), and IL-6 (*P* < 0.001) levels (Figures 3(d)–3(f)).

In Table 3, it showed that antibiotic use might be appropriate when PCT > 1 ng/mL or CRP > 40 mg/mL, compared with patients who did not change antibiotics. In our study, it was found that elevated IL-6 levels may not be desirable as an indication of the need for antibiotic treatment; however, IL‐6 > 60 pg/mL (*P* = 0.527) could not be suggested the failure of initial broad-spectrum antibiotic treatment.

## 4. Discussion

Anthonisen et al. proposed that the criteria most commonly used to diagnose acute exacerbations of COPD (AECOPD) were mostly based on patient subjective symptoms [22]. The aggravation of COPD has a profound impact on the patients' health, functional capacity, and lung function. Data from the study with more than 73,000 patients showed that less than half of the patients hospitalized due to exacerbation of COPD survived for another 5 years [23]. Combined with the results of laboratory tests and the patients' condition were evaluated and given a reasonable antibiotic prescription. And the use of antibiotics could be reduced without adverse effects on patients.

There were 325 patients with AECOPD in the study; 264 of the patients (81.2%) were given antibiotics in the early stages of treatment. Whether these antibiotic treatments were necessary had aroused our concern. In our study, we excluded patients with bronchial asthma (critical type) on the grounds that they were treated with systemic glucocorticoids after admission because of their anti-inflammatory properties [24]. The presence of these conditions was likely to have influenced our observation in the effects of antibiotic use. Patients with clear evidence of infectious diseases such as bronchiectasis, sepsis, and bacteremia were not included in the study. These further reduced the interference factors.

In our study, the levels of PCT, CRP, and IL-6 in patients with AECOPD were all higher than those of patients with COPD (*P* < 0.05). The specificity of PCT (93.5%) was better, but the sensitivity was only 34.5%. The sensitivity (56.3%) and specificity (91.3%) of CRP were both lower than PCT. The AUC (0.773) and sensitivity (71.7%) of IL-6 were the highest, but the specificity (78.3%) was the lowest among the three indicators. It was suggested that we should diagnose the patient with AECOPD, combining the clinical manifestations of the patients and the expression levels of PCT and IL-6.

Previous studies have shown that objectively laboratory indicators such as PCT and CRP have a good directive function, both in guiding antibiotic therapy and diagnosis of AECOPD [25]. Research by Butler et al. showed that patients with AECOPD were less likely to be prescribed antibiotics under the guidance of the CRP test, and there was no adverse evidence compared with patients in the routine care group in primary care clinics [20]. Compared with CRP, PCT showed a stronger suggestive effect on the severity of bacterial infection [26]. Some studies have produced algorithms for the use of calcitonin to guide antibiotic use based on the type of acute respiratory infection [27]. These included community-acquired pneumonia, bronchitis, exacerbations of chronic obstructive pulmonary disease or asthma, septicaemia, and respiratory infections. The consensus algorithm of PCT recommends/mandates cessation of antibiotic use when PCT ≤ 0.25/0.1 ng/L [27]. Bartoletti et al. believed that the initiation or escalation of antibiotic therapy should not be based solely on PCT serum levels. In order to interpret the PCT results correctly, clinical and radiological findings should be taken into account to facilitate the assessment of disease severity and patient conditions [28].

Our results indicated that the specificity (65.5%) and sensitivity (85.2%) of CRP were superior in patients who also received antibiotic treatment, depending on whether they changed antibiotics or not in case control 2. Logistic result analysis indicated that CRP > 20 mg/mL was more needed for antibiotic treatment and could require advanced antibiotic treatment; CRP > 40 mg/mL should do so. However, we found that even in patients diagnosed with AECOPD with failure of initial antibiotic treatment, the PCT level of 7 patients (11.5%) still was 0.25-1 ng/mL, and its level of more than 1 ng/mL could be found in another 11 patients. It suggested that PCT elevation was not significant even in patients who had a failed initial antibiotic treatment. Combined with the ROC curve results in case 1, the AUC of PCT was 0.647. We believed that PCT was less sensitive in the diagnosis of AECOPD; at the same time, IL-6 was more sensitive; CRP was better in guiding antibiotic treatment. Llor et al. also found that the antibiotic treatment effect of AECOPD patients was better, and the amount of antibiotic prescriptions was reduced when the CRP test was used to guide antibiotic treatment in a cross-sectional survey of 6 primary medical institutions [29].

Other biomarkers associated with inflammation had been reported to guide antibiotic therapy. IL-6 was a proinflammatory cytokine that had been implicated in a complex series of chronic diseases [30]. IL-6 signal occurs in two different ways, classical signal and transsignal [31, 32]. In IL-6 transsignal transduction, the soluble form of IL-6 receptor (SIL-6R) is widely expressed in lung cells, since SIL-6R could trigger IL-6 signal transduction in cells that normally do not respond to IL-6. Transsignal transduction was associated with the pathogenesis of many inflammatory diseases, and IL-6 was involved in airway remodeling in COPD patients [33]. It was also shown that a high level of IL-6 was associated with long-term mortality and poor physical condition in patients with COPD [34]. To our knowledge, studies on IL-6 application and guidance of AECOPD antibiotic treatment were limited. Our retrospective study found that the AUC of IL-6 predicted antibiotic replacement was 0.771. It was slightly higher than PCT (AUC = 0.764) and CRP (AUC = 0.764), and its sensitivity (80.3%) was slightly higher than PCT (75.4%). The specificity of IL-6 (60.6%) was the lowest among the three indicators. This suggested that IL-6 was slightly less valuable compared with CRP or PCT in evaluating antibiotic therapy in AECOPD patients.

During the study, we found that only a small number of patients had positive sputum culture results, which did not rule out the possibility of bacterial colonization. Clinicians paid more attention to laboratory results for bacterial infections and ignored relevant tests for viruses, resulting in fewer positive virus tests. The use of virus and bacterial testing to guide AECOPD treatment was limited by the long cycle and imitated detection methods of a virus. In view of the minority number of positive microbiological and hierological results, no statistical analysis was performed in this study. On the one hand, the blood sample results of patients were collected immediately after admission. During the treatment, clinical manifestations and related indicators of patients would also change as the disease progression. However, we only analyzed the examination results of PCT, CRP, and IL-6 of patients at the beginning of admission. On the other hand, some patients might receive treatment prior to admission and may have been further admitted due to poor treatment outcomes. We retrospectively analyzed the value of PCT, CRP, and IL-6 levels in antibiotic use in patients with AECOPD. Not all of the data we collected might be in the initial stage of the acute episode of sCOPD; this process might lead to errors in our results. In the later study, we will further analyze the dynamic changes of CRP, PCT, and IL-6 during the treatment of AECOPD patients.

## 5. Conclusion

There are no objective indicators of when AECOPD patients need antibiotic therapy. In this study, we found that elevated IL-6 and PCT have higher diagnostic value for AECOPD. CRP has a high diagnostic value for antibiotic use in AECOPD patients. In order to further understand the development of the disease, we hope that we can find more appropriate blood markers to guide antibiotic use in patients with AECOPD.

## Figures and Tables

**Table 1:** Demographic features and laboratory findings of the patients.

	sCOPD	*n* = 46	AECOPD	*n* = 325	*P* value
Male	22	47.8	200	61.5	0.076
Age (age)	69.8 ± 12		72.62 ± 11.69		0.522
Length of stay (days)	7.89 ± 3.33		9.32 ± 5.28		0.117
In intensive care	0		35	10.8	<0.001
Symptom					
Cough	38	82.6	321	98.8	<0.001
Expectoration	35	76.1	320	98.5	<0.001
Shortness of breath	12	26.1	113	34.8	0.244
Wheezes	11	23.9	88	27.1	0.679
Chest tightness	20	43.5	121	37.2	0.406
Fever	7	15.2	85	26.2	0.108
Chest pain	2	4.4	23	7.1	0.706
Dyspnea	0		24	7.4	0.113
Purulent sputum	5	10.9	110	33.9	<0.001
Diabetes	14	30.4	65	20	0.106
Coronary heart disease	9	19.6	101	31.1	0.11
Hypertension	28	60.9	187	57.6	0.551
Cerebral infarction	9	19.6	63	19.4	0.977
Bronchodilation	4	8.7	41	12.6	0.446
Bronchial asthma	16	34.8	72	22.2	0.059
Pulmonary embolism	0		16	4.9	0.25
Malignant tumor	0		9	2.8	0.528
PCT (ng/mL)	0.04 ± 0.05		0.19 ± 0.60		0.005
CRP (mg/mL)	3.91 ± 4.99		28.92 ± 41.93		<0.001
IL-6 (pg/mL)	4.68 ± 4.64		37.14 ± 63.20		<0.001
Lung CT indicated infection	14	30.4	223	68.6	<0.001
Abnormal pulmonary auscultation	12	26.1	249	76.6	<0.001
Antibiotic used	12	26.1	264	81.2	<0.001
Course of use of the first antibiotic (days)	1.22 ± 2.31		3.86 ± 2.40		0.24
The total course of antibiotics (days)	1.28 ± 2.5		5.44 ± 4.21		0.52
Changed antibiotics	0		61	18.8	0.001
Antibiotic step-down therapy	1	2.2	35	10.8	0.115
Combined other anti-gram-negative bacilli antibacterial drugs	0		3	0.9	0.822
Combined other anti-gram-positive cocci antibacterial drugs	0		9	2.8	0.528
Add antifungal drugs	0		57	17.5	0.002
Add antiviral drugs	0		2	0.6	0.588
Intravenous glucocorticoids	0		74	22.8	<0.001

**Table 2:** Evaluation of the diagnostic and predictive value of the antibiotic prescription value of AECOPD inflammatory factors using ROC curve.

Group	Index	AUC	*P* value	95% CI	Cut-off value	Sensitivity	Specificity
Case 1	PCT	0.647	0.001	0.573-0.720	0.065	34.5	93.5
	CRP	0.764	<0.001	0.703-0.814	8.210	56.3	91.3
	IL-6	0.773	<0.001	0.738-0.843	5.262	71.7	78.3

Case 2	PCT	0.764	<0.001	0.675-0.810	0.055	75.4	65.5
	CRP	0.764	<0.001	0.640-0.786	20.205	85.2	65.5
	IL-6	0.771	<0.001	0.679-0.821	17.835	80.3	60.6

**Table 3:** Prediction of PCT, CRP, and IL-6 levels in AECOPD patients with antibiotic replacement therapy.

Variables	No-changed	%	Changed	%	*P* value	B	S.E.	Wald	*P*	OR	OR (95% CI)
	*n* = 203		*n* = 61								
PCT (ng/mL)	0.12 ± 0.29		0.47 ± 0.94		<0.001						
CRP (mg/mL)	25.69 ± 37.52		60.10 ± 53.75		<0.001						
IL-6 (pg/mL)	27.96 ± 39.56		92.99 ± 95.41		<0.001						
PCT (ng/mL)											
<0.25	191	94.1	43	70.5	<0.001			3.98	0.137		
0.25-1	7	3.4	7	11.5	0.014	0.375	0.65	0.34	0.563	1.456	0.408-5.191
>1	5	2.5	11	18.0	<0.001	1.314	0.66	3.98	0.046	3.722	1.023-13.537
CRP (mg/mL)											
<20	132	65.0	10	16.4	<0.001			20.24	<0.001		
20-40	31	15.3	16	26.2	<0.001	1.935	0.45	18.47	<0.001	6.923	2.864-16.732
>40	40	19.7	35	57.4	<0.001	1.808	0.63	8.19	0.004	6.097	1.768-21.023
IL-6 (pg/mL)											
<60	174	85.7	31	50.8	<0.001						
≥60	29	14.3	30	49.2	<0.001	0.413	0.65	0.4	0.527	1.511	0.421-5.432
Constant						-2.596	0.33	62.43	<0.001	0.075	

## Data Availability

The data used to support the findings of this study are available from the corresponding author upon request.
